# Platelet response during the second cycle of decitabine treatment predicts response and survival for myelodysplastic syndrome patients

**DOI:** 10.18632/oncotarget.3914

**Published:** 2015-04-23

**Authors:** Hyun Ae Jung, Chi Hoon Maeng, Moonjin Kim, Sungmin Kim, Chul Won Jung, Jun Ho Jang

**Affiliations:** ^1^ Division of Hematology-Oncology, Department of Medicine, Samsung Medical Center, Sungkyunkwan University School of Medicine, Seoul, Korea; ^2^ Division of Hematology-Oncology, Department of Medicine, Hallym University Medical Center, Hallym University College of Medicine, Dontan, Korea; ^3^ Division of Hemato-Oncology, Department of Internal Medicine, Kyung Hee University Medical Center, Seoul, Korea

**Keywords:** myelodysplastic syndrome, platelet

## Abstract

Despite the efficacy of decitabine to myelodysplastic syndrome (MDS), there is a wide range of responses, and no definite predictive marker has been identified. This study aimed to describe the efficacy of decitabine and to identify potential predictors of response and survival in patients with MDS. We retrospectively analyzed clinical data of MDS patients at Samsung Medical Center between August 2008 and August 2011. The response assessment was conducted using the International Working Group (IWG) response criteria for MDS. We analyzed 101 MDS patients (total 613 cycles) who received decitabine for a median of four cycles. The overall response was 52.5% (*n* = 53/101). The median time to any response was two cycles with the median overall survival of 16.7 months. Patients who showed hematologic improvement had significantly longer survival than those who did not (9.8 vs. 22.9 months, *p* = 0.004). The difference in OS was evident in the Intermediate-2/High risk group (*p* = 0.002) but not in the Intermediate-1 risk group (*p* = 0.145). Multivariate analysis confirmed that platelet response (no platelet transfusions for at least 3 days) during the second cycle of treatment was an independent predictor for response, OS and Leukemia free survival. Based on the results of this study, for patients with hematological improvement, recovery of platelet count by the second cycle of therapy can be used as an early predictive marker of improved survival and an increased response rate.

## INTRODUCTION

Myelodysplastic syndromes (MDS) are a group of clonal hematopoietic disorders marked by ineffective hematopoiesis, peripheral cytopenias, and an increased risk of transformation to acute myeloid leukemia. MDS is associated with a wide array of clinical manifestations and treatment outcomes.

MDS treatment is based on prognostic factors that predict survival and likelihood of progression to AML. Currently, development of prognostic systems that allow for risk stratification and that can guide the timing and choice of MDS therapy is needed. The International Prognostic Scoring System (IPSS) remains the most widely used prognostic system for therapeutic decisions. The IPSS advises the use of supportive care with transfusion and growth factor supplementation as treatment in lower risk groups of patients. Allogeneic stem cell transplantation is the only curative treatment for the high risk group; however, in the post-epigenetic therapy era, treatment approaches for patients with MDS have improved significantly. Specifically, hypomethylating agents improve the transfusion requirement and quality of life while decreasing leukemic transformation and survival. Azacitidine [1-3] and decitabine [4-6] are currently the two available agents with the capacity to induce DNA hypomethylation in vivo. Recently, DNA methyltransferase inhibitors have become the mainstay of MDS treatment since being approved in the United States and Europe for the treatment of higher risk MDS (i.e., intermediate-2 and high risk, according to the IPSS guidelines). Azacitidine has been studied in higher-risk MDS patients in two randomized multicenter trials, which include Cancer and Leukemia Group B (CALGB) 9221 [7] and AZA-001 [1]. Decitabine, a DNA-methyltransferase inhibitor, has a wide range of overall response rates from 30%-60% based on literature published in Western countries [4, 8-10] and Korea [11]. However, the non-response rate for hypomethylating agents is high. Additionally, there are few studies investigating the treatment response in Asian populations, and there is no known predictive marker for response to decitabine therapy. It is known that pretreatment risk stratification is related to MDS patient survival outcome. Therefore, we aimed to assess the efficacy of decitabine and to identify predictors of response to decitabine therapy.

## RESULTS

### Patient characteristics

We identified 101 consecutive patients who received decitabine as first-line treatment for MDS and had available clinical and pathologic data between January 2008 and December 2011. Patient demographics are described in Table 1. The median age was 65 years (range, 18-84 years old), and 30% of the patients were women. There was no significant difference in the baseline characteristics of responding and non-responding patients.

**Table 1:** Patient characteristics

Characteristic	Total N = 101	Non-responsive	Responsive	p–value
Age (years)				0.234
65 or less	48(47.5%)	27(56.3%)	21 (43.8%)	
Over 65	53(52.5%)	23(43.4%)	30(56.6%)	
Sex				0.561
Male	71(70.3%)	35(49.3%)	36(50.7%)	
Female	30(29.7%)	15(50%)	15(50.0%)	
WHO subtype				0.771
RA/RARS/RDS-U	5(5.0%)	2(40.0%)	3(60.0%)	
RCMD/RCMD-RS	27(26.7%)	16(59.3%)	11(40.7%)	
RAEB-1	21(20.8%)	11(52.4%)	10(47.6%)	
RAEB-2	32(31.7%)	14(43.8%)	18(56.3%)	
CMML	16(15.8%)	7(43.8%)	9(56.3%)	
WPSS risk category				0.731
Very low/Low	10(9.9%)	5(50.0%)	5(50.0%)	
Intermediate	13(12.9%)	8(61.5%)	5(38.5%)	
High	43(42.5%)	19(44.2%)	24(55.8%)	
Very high	19(18.8%)	11(57.9%)	8(42.1%)	
IPSS risk category				0.604
Low	7(6.9%)	5(71.4%)	2(28.6%)	
INT-1	45(44.6%)	20(44.4%)	25(55.6%)	
INT-2	38(37.6%)	19(50.0%)	19(50.0%)	
High	11(10.9%)	6(54.5%)	5(45.5%)	
IPSS cytogenetic risk category				0.130
Good	52(51.5%)	20(38.5%)	32(61.5%)	
Intermediate	24(23.8%)	18(75.0%)	6(25.0%)	
High	25(24.8%)	12(48.0%)	13(52.0%)	

### Treatment response

A total of 613 cycles of decitabine treatment were administered in 101 patients with MDS. The median follow-up period was 20.7 months (range: 1.0-89.6 months). The median number of decitabine treatment cycles was four (range: 1-34 cycles). The median number of cycles to any response was two (range: 1-17 cycles), and the median time to best response was 2.3 months (Table 6). Of the patients who received decitabine, the overall response rate (ORR, Complete response(CR), Partial response(PR), Marrow complete response(mCR) and Hematologic improvement(HI)) was observed in 51 of 101 patients (50.5%) (Table 2). Among the patients that responded to decitabine treatment, 37 (72.5%) showed a treatment response within two cycles of treatment, and 14 patients (27.5%) showed a treatment response after two cycles of decitabine treatment (Table 3). Platelet response during decitabine treatment was indicated in 48 patients (47.5%), most of whom demonstrated a response (N = 31, 64.6%) within two cycles of decitabine therapy.

**Table 2:** Decitabine treatment response

Response	No. of patients (%) N=101
CR+PR	17(16.8%)
m CR with HI	9(8.9%)
m CR without HI	7(6.9%)
HI only	18(17.8%)
SD	14(13.9%)
Failure	36(35.6%)
CR+PR+m CR	33(32.7%)
CR+PR+m CR+HI	51(50.5%)
CR+PR+m CR+ HI +SD	65(64.4%)
HI-N	30(29.7%)
HI-E	39(38.6%)
HI-P	48(47.5%)

CR, Complete response; PR, Partial response; HI, Hematologic improvement; SD, Stable disease; HI-N, Hematologic improvement in neutrophils; HI-E, Hematologic improvement in erythroids; HI-P, Hematologic improvement in platelets.

**Table 3:** Decitabine treatment time to response

	Median no. of decitabine cycles (range)
Time to any response	2(1-17)
Time to hematologic response	2(1-17)
Time to any HI	2(1-17)
Time to HI-N	6 (1-13)
Time to HI-E	3(1-17)
Time to HI-P	2(1-13)

HI-N, Hematologic improvement in neutrophils; HI-E, Hematologic improvement in erythroids; HI-P, Hematologic improvement in platelets.

### Predictive factors of treatment response and survival

**Figure 1:**
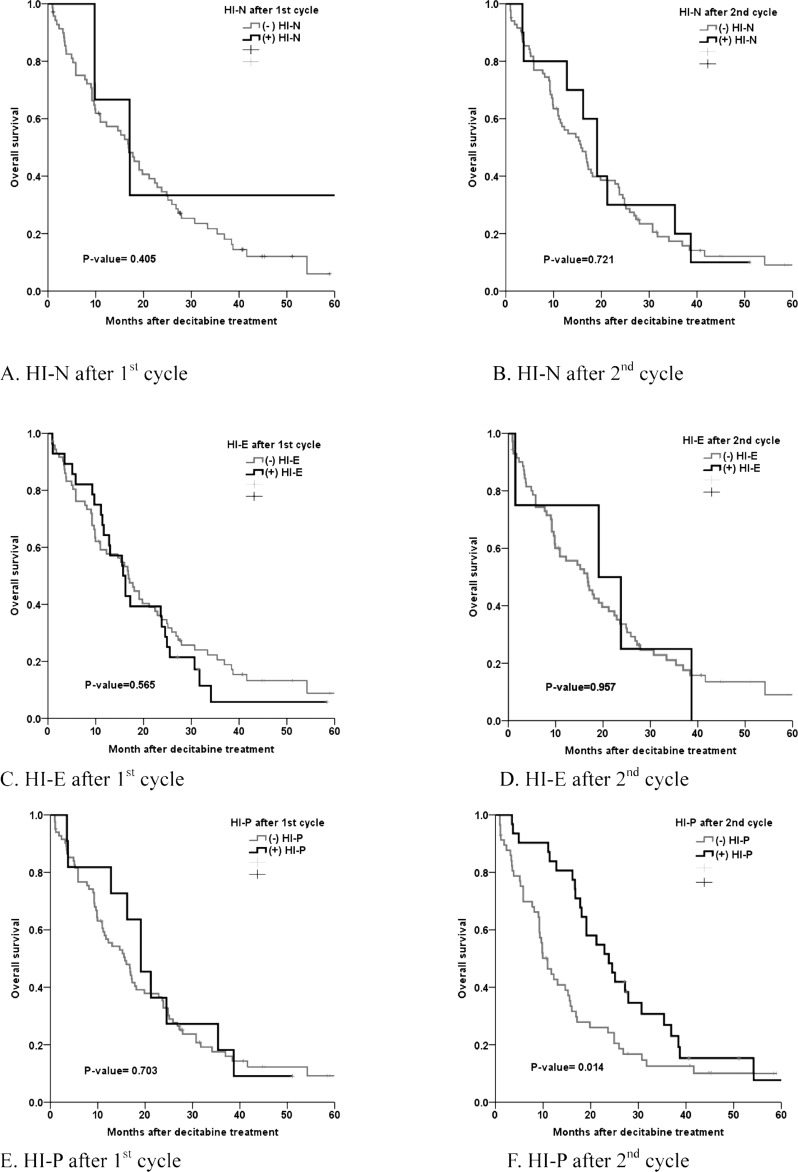
Hematologic improvement and OS **A.** HI-N after first cycle. **B.** HI-N after second cycle **C.** HI-E after first cycle **D.** HI-E after second cycle **E.** HI-P after first cycle **F.** HI-P after second cycle.

The median overall survival duration in all the patients was 16.7 months (range, 0.9-60.8 months). Univariate analysis showed that IPSS, the WHO classification-based Prognostic Scoring System (WPSS) and the platelet response during the second cycle of decitabine all significantly predicted favorable ORR, OS and LFS (Table 4, Table 5 and Figure 1).

**Table 4:** Prognostic factors of overall response and overall survival

	OS (months)	*p*-value	LFS (months)	*p*-value
Age (years)		0.871		0.367
65 or less	16.0		9.8	
Over 65	16.7		16.2	
Sex		0.361		0.307
Male	16.9		16.2	
Female	12.8		11.2	
WHO subtype		0.130		0.170
RA/RARS/RDS-U	20.5		14.0	
RCMD/RCMD-RS	19.4		22.4	
RAEB-1	17.7		3.8	
RAEB-2	16.0		10.8	
CMML	13.8		11.2	
WPSS risk category		0.04		0.06
Very low/Low	42.4		42.4	
Intermediate	38.6		24.0	
High	19.8		21.2	
Very high	10.9		8.5	
IPSS risk category		0.024		0.003
Low	30.7		22.1	
INT-1	19.1		21.2	
INT-2	12.8		9.0	
High	10.9		5.3	
IPSS cytogenetic risk category		0.415		0.438
Good	19.1		21.2	
Intermediate	11.7		8.5	
High	15.7		12.1	

LFS, Leukemia-free survival; OS, Overall survival.

**Table 5:** HI and ORR/OS

	ORR (%)	*p*-value	OS (months)	*p*-value	LFS (months)	*p-value*
First cycle						
HI-N		0.114		0.405		0.951
No	47.9		16.8		14.0	
Yes	52.1		17.8		14.0	
HI-E		0.742		0.565		0.912
No	51.1		16.9		12.1	
Yes	50.0		15.7		11.5	
HI-P		0.079		0.703		0.705
No	46.4		15.7		12.1	
Yes	62.1		19.1		16.2	
Second cycle						
HI-N		0.250		0.721		0.903
No	44.4		15.7		12.1	
Yes	75.0		19.1		17.5	
HI-E		0.079		0.957		0.976
No	47.1		16.7		11.2	
Yes	71.8		19.1		17.5	
HI-P		<0.001		0.014		0.019
No	26.4		10.9		7.1	
Yes	86.1		23.2		18.1	

ORR, Overall response rate; HI-N, Hematologic improvement in neutrophils; HI-E, Hematologic improvement in erythroids; HI-P, Hematologic improvement in platelets.

**Figure 2:**
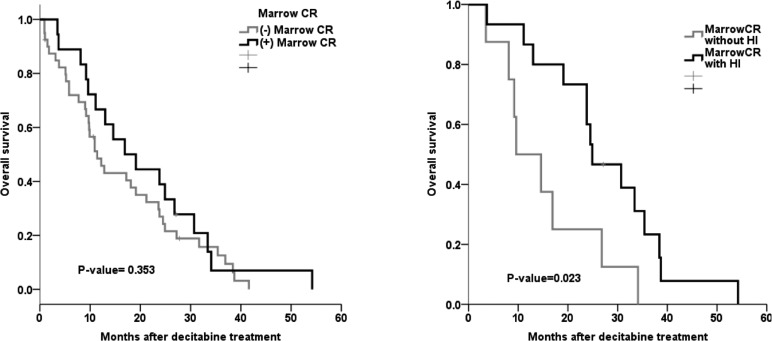
m-CR and OS in patients with BM blasts >5%

**Figure 3:**
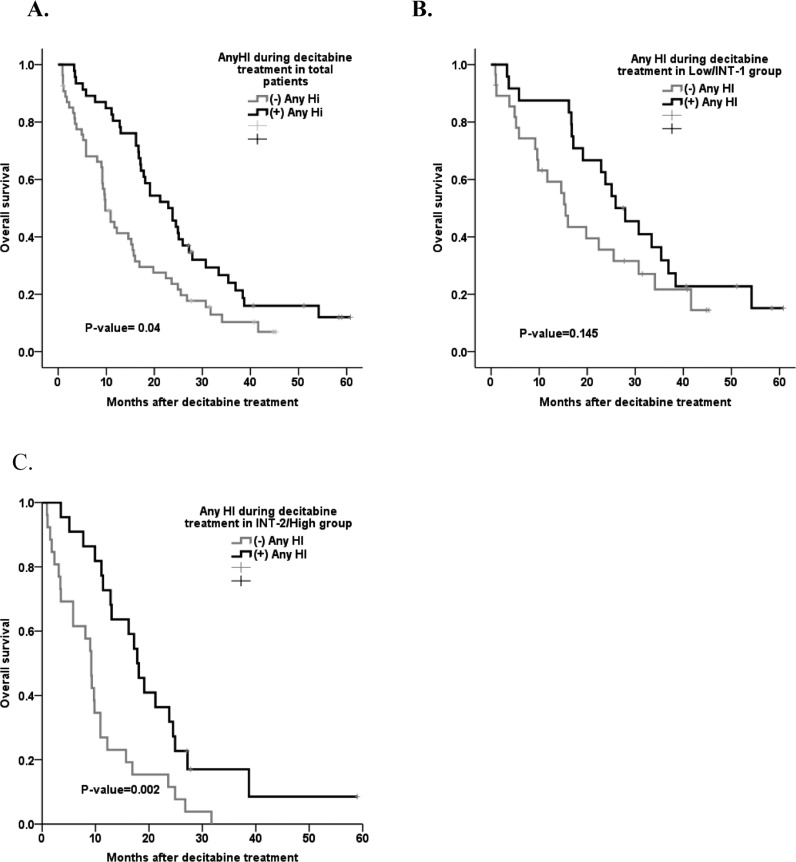
Survival curve based on the presence of hematologic improvement during treatment **A.** Any HI in all patients during decitabine treatment (*p*-value = 0.040). **B.** Any HI in Low risk (IPSS INT-1/Low) patients during decitabine treatment (*p*-value = 0.145). **C.** Any HI in High risk (IPSS INT-2/High) patients during decitabine treatment (*p*-value =0.002).

Figure 2 shows the relationship between m-CR and OS in patients with bone marrow blasts >5%. The OS in patients with marrow complete remission (m-CR) was not significantly different compared to that of patients without m-CR (*p*-value = 0.353). However, the patients who showed hematologic improvement (HI) had a significantly higher survival rate than those who did not (11.8 vs. 23.8 months, *p* = 0.001) (Table 6.). The median OS in patients without HI during or after decitabine treatment was 9.8 months, and the median OS in patients with HI was 22.9 months (*p*-value = 0.004, Figure 3). The difference in OS was evident in the Intermediate-2/High risk group (*p*-value = 0.002) but not in the Intermediate-1 risk group (*p*-value 0.145, Figure 3).

**Table 6:** Relationship between mCR and OS in patients with BM blasts >5%

		Median OS (months)	*p-*value	
Marrow CR (+)		21.2	0.023	
	Presence of HI	9.6		
	Absence of HI	24.9		

Multivariate analysis indicated that the platelet response during the second cycle of decitabine treatment significantly predicted ORR, OS and LFS (Table 7).

**Table 7:** Multivariate analysis for ORR, OS and LFS

	ORR			OS			LFS		
	Exp (B)	*p*-value	95% CI	Exp (B)	*p*-value	95% CI	Exp (B)	*p*-value	95% CI
Second cycle HI-P	25.6	<0.001	7.42-91.0	0.58	0.031	0.36-0.95	0.54	0.014	0.33-0.88
IPSS (Low vs. High)	2.2	0.05	0.974-4.73	1.35	0.058	0.96-1.84	1.41	0.025	1.04-1.92

95% CI, 95% confidence interval.

## DISCUSSION

The results of this study suggest that the platelet response that occurs during the second cycle of decitabine can predict the overall response and survival of MDS patients.

Currently, genetic oncology faces the challenge of identifying patients who will be benefit from target agents. Therefore, biomarkers that can guide clinical decisions and predict the response to treatment are needed. Additionally, identifying patients who will derive the greatest clinical benefit from hypomethylating agents remains a challenge; however, this could be resolved by identifying biomarkers that predict patient response.

Unlike conventional chemotherapy, several cycles of treatment with a hypomethylating agent are often needed to appreciate the effect of the therapy. In both the CALGB 9221 and AZA-001 azacitidine trials, several courses of therapy (four to six cycles) were required to achieve a response. In a small portion of patients, responses could be observed after up to 12 cycles of therapy. Therefore, the ability to predict response to treatment at an earlier time is needed.

There exist very few studies about hypomethylating agent predictive markers in MDS patients. In previous studies, pretreatment risk stratification was based on IPSS or WPSS, and initial LDH level was beneficial for OS in patients treated with azacitidine [12, 13]. Previous low dose ara-C treatment, bone marrow blasts >15%, poor performance and more than four units of red blood cells transfused every eight weeks were factors that predicted a lower response rate to 5-azacitidine [14]. In addition, some reports demonstrated that specific cytogenetic abnormalities may be associated with a better response to hypomethylating agents [15, 16]. Recently, specific molecular mutations in the methylation pathway have been identified, including *TET*2, *DNMT3A* and *IDH/IDH2* [17-19]; however, these methylation machinery genes were detected in only 10-30% of MDS patients [20, 21].

Lee et al. [11] demonstrated that decitabine treatment prolonged OS in patients that achieved hematologic improvement. Previous randomized trials reported significant prolongation of OS or LFS in higher risk MDS patients receiving hypomethylating agents, but these agents did not lower the disease risk [4]. Similarly, our results showed that the patients who showed HI exhibited significantly increased survival compared to patients who did not show HI. The patients who showed hematologic improvements (HI) had significantly longer survival rates than those who did not (11.8 vs 23.8 months, *p* = 0.001) (Table 6.) In comparison, the OS in patients with mCR was not statistically different compared to that of those without m-CR. Specifically, we noted that HI was achieved and indicated by the platelet response.

Few reports have analyzed platelet response as a predictive factor of patient survival [22, 23]. Decitabine has a 20%-50% response rate for thrombocytopenia in MDS patients. Decitabine enhances normal megakaryocyte outgrowth and differentiation of normal megakaryocytes into platelets [24]. Platelet level has often been observed as the first response to treatment, whereas red cell count and neutrophilic granulocyte count respond later during therapy. The neutrophil response may be delayed due to the slow disappearance of blasts from the bone marrow [23]. Hypomethylation of genes important for megakaryopoesis may be functionally linked by 5-aza-2′-deoxycytidine. This hypothesis is based on the observed hypermethylated promotor region of the p15 tumor suppressor gene [22]. Liekee et al. reported that a two-fold increase in platelet count after the first cycle of azacitidine treatment predicted longer OS and may be a useful early indicator of favorable azacitidine treatment outcome [23] in MDS and AML patients. The results indicated that 16% of patients with MDS and AML had an increased platelet count after the first cycle of azacitidine, which was associated with a significantly better OS rate. However, in this study, only seven MDS patient who received azacitidine had an increased platelet count. In our study, most MDS patients (N=31/48, 64.6%) showed an increased platelet count within two cycles of decitabine. By the second cycle, the platelet response was a significant predictive factor for OS and LFS after adjusting for known predictors (IPSS).

Our study was limited because of its retrospective nature. Additional studies that include a larger number of patients treated homogenously with hypomethylating agents are needed for external validation. In the future, we will use Sanger sequencing to investigate mutations in methylating machinery genes (TET2 and DNMT3A) in patients who receive decitabine as a first-line treatment. Two hypomethylating agents are currently available. However, only azacitidine has been shown to be associated with prolonged survival in prospective study until now. In our study, patients received decitabine treatment. Hypomethylating agents are considered today as the first line treatment for MDS patients classified as INT-2 and High-risk IPSS. However, in this study, 49.8% of the patient population are classified as Low and INT-1. In this respect, our study has limitation.

In conclusion, decitabine is effective (ORR 50.5%) and can cause a rapid platelet response that is apparent by the second treatment cycle in MDS patients. Overall survival is significantly longer in patients with hematologic improvement. Based on the results of this study, for patients with hematological improvement, recovery of platelets by the second cycle of therapy can be used as an early predictive marker of improved survival and an increased response rate.

## MATERIALS AND METHODS

### Patients

We retrospectively analyzed the medical records of patients who were diagnosed with MDS (de novo or secondary) based on the World Health Organization (WHO) classifications. This study was approved by the Samsung Medical Center Institutional Review Board.

### Treatment

Decitabine was given intravenously over one hour at a dose of 20 mg/m^2^ daily for five consecutive days, and each course was repeated every four weeks. All patients received decitabine treatment as first-line treatment. Physicians were advised to continue decitabine treatment for at least four courses unless a patient either died, experienced unacceptable adverse events, or withdrew from the study. Bone marrow examination was conducted with every two courses until complete remission (CR) was confirmed. Prophylactic antimicrobials, hematopoietic growth factors and other supportive care were administered at the discretion of the treating physician.

### Response criteria

The response assessment was conducted using the International Working Group (IWG) response criteria for myelodysplasia [25]. The hematologic response was evaluated during each cycle, and patients who were classified as high or intermediate-2 according to the IPSS underwent a bone marrow biopsy to evaluate their response to treatment. A complete response (CR) required the disappearance of all signs and symptoms, a bone marrow biopsy with less than 5% blasts, peripheral blood count with an absolute neutrophil count of 1×10^9^/L or more, and a platelet count of 100×10^9^/L or more. A marrow-CR (mCR) was defined as a bone marrow blast less than 5% that did not meet peripheral blood count criteria. A partial response (PR) was defined as a reduction in the blast count to less than 50% but more than 5%. Response criteria for CR and PR required a treatment duration of at least four weeks. All HI was determined based on the modified IWG criteria. The HI are measured in patients with pretreatment abnormal values: hemoglobin level less than 110 g/L (11g/dL) or RBC-transfusion dependence, platelet count less than 100 × 10^9^/L or platelet-transfusion dependence, absolute neutrophil count (ANC) less than 1.0 × 10^9^/L. Pretreatment baseline measures of cytopenias are averages of at least 2 measurements (not influenced by transfusions, ie, no RBC transfusions for at least 1 week and no platelet transfusions for at least 3 days) over at least 1 week prior to therapy.

Major and minor HI must last for at least 8 weeks. An overall response rate (ORR) was defined according to CR, PR, mCR and HI. Overall survival was measured from the date of initiation of decitabine therapy to the date of death or the last follow-up visit. Leukemia-free survival was measured from the date of initiation of decitabine therapy to the date of death or leukemic transformation.

### Statistical analysis

Patient characteristics were compared using the Chi-square and Fisher's exact test (categorical variables) between responsive and non-responsive patients who received decitabine treatment. Survival time was estimated using the Kaplan-Meier method, and statistical differences were compared using log-rank analysis. Multivariate analysis was performed using stepwise Cox proportional hazards regression modeling to assess the independent prognostic role of each clinicopathologic variable in OS and LFS. A stepwise multiple logistic regression was used to determine ORR. Statistical analyses were performed using SPSS 19.0 (SPSS Inc., Chicago, IL), and statistical significance was considered at *p* ≤ 0.05.
